# The Computerized Developmental Eye Movement (DEM) Test: Normative Data for School-Aged Children

**DOI:** 10.3390/vision8030047

**Published:** 2024-08-09

**Authors:** Daniela Protasevica, Evita Kassaliete, Anete Klavinska, Madara Alecka, Asnate Berzina, Viktorija Goliskina, Marija Koleda, Rita Mikelsone, Elizabete Ozola, Tomass Ruza, Evita Serpa, Aiga Svede, Daniela Toloka, Sofija Vasiljeva, Liva Volberga, Ilze Ceple, Gunta Krumina

**Affiliations:** Department of Optometry and Vision Science, Faculty of Physics, Mathematics and Optometry, University of Latvia, LV-1586 Riga, Latvia; anete.klavinska@gmail.com (A.K.); madaraaleckaa@gmail.com (M.A.); asnate.berzina@gmail.com (A.B.); vika.golishkina@gmail.com (V.G.); eklisia21@gmail.com (M.K.); ritamikelsone2@gmail.com (R.M.); elizabete.ozola0@gmail.com (E.O.); tomass.ruza@gmail.com (T.R.); evita.serpa@lu.lv (E.S.); aiga.svede@lu.lv (A.S.); tolokadaniela@gmail.com (D.T.); vasakini@yahoo.com (S.V.); livavolberga@gmail.com (L.V.); ilze.ceple@lu.lv (I.C.); gunta.krumina@lu.lv (G.K.)

**Keywords:** school-aged children, DEM test, fixations, reading disorders

## Abstract

The aim of the study was to determine the normative data of the computerized DEM test for school-age children in Latvia. The study analyzed data on the performance (test execution time, duration, number of fixations, and number of errors) of 291 children while completing the computerized DEM test. Eye movement fixations were recorded with a Tobii Pro Fusion video-oculograph (250 Hz). According to the results of the study, the performance of the computerized DEM test is 77 %. For the study, 1 SD (one standard deviation) was chosen as a criterion for determining test norms. In the study, the norms of the computerized DEM test in Latvia were developed in class groups—from 1st to 6th grade (aged 7 to 12 years), the results were summarized in a table as the minimum performance values of the computerized DEM test.

## 1. Introduction

In today’s society, literacy is one of the most important life skills involved in virtually all daily activities. As a result, poor literacy affects all aspects of a person’s life [1]. Therefore, it is a current challenge to perform a precise assessment of reading skills and determine the possible causes of reading disorders.

**Figure 1:**
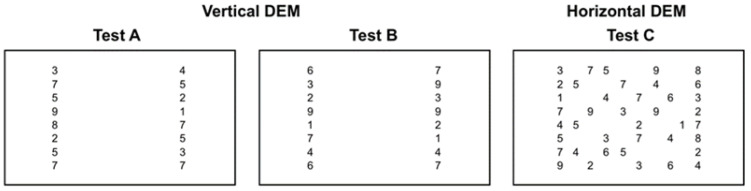
The three cards of the DEM test: (A–C). The first two cards (A,B) comprise two columns of 20 numbers for each card. Card C is composed of the same 80 numbers from the A and B cards but arranged in a horizontal pattern, similarly to a reading text. The test is a practical paper-based psychometric test designed for the assessment of ocular movement in a reading-like condition [2,4]. Adapted from [5].

The Developmental Eye Movement (DEM) (see Figure 1) test is a visually verbal number-naming test designed for children aged 6 to 13, with or without learning disabilities, or suspected of having learning disabilities. This test is used to assess children’s eye movements, especially saccadic movements while reading numbers aloud from the test cards [2]. It is an oculomotor test that has a specific method to distinguish between naming problems and oculomotor disorders [2,3,4].

Currently, the norms of the paper version of the DEM test have been developed and are available for nine languages: English, Portuguese, Spanish, Italian, Mandarin, Cantonese, Japanese, French, and Latvian [2]. However, there is very little research on the computerized version of the DEM test. By recording eye movements during the computerized DEM test, it is possible to analyze other objective DEM test parameters and develop new normative values that cannot be assessed with the paper version of the test. Based on an objective assessment of eye movements using eye-tracking, it has been concluded that the average fixation duration is longer when reading the DEM test A and B cards comparing to the C test card [6]. A correlation is also observed between the average fixation duration and the total reading time of the DEM test C text [7]. Tanke and colleagues [5] explored the completion times of the computerized DEM test cards across different age groups and concluded that the DEM test is not suitable for measuring saccade behavior but can be a useful indicator of visual–verbal naming skills, visual processing speed, and other cognitive factors of clinical relevance and it does not detect oculomotor problems even for children with Nystagmus [8]. However, clear, normative values for the computerized DEM test and the eye movement parameters that can be assessed during the test have not been established.

The development of computerized DEM test norms is an investment in the development of new technologies to make the computerized DEM test an accessible and widely used tool for visual processing speed and eye movement assessment.

The aim of the current study is to establish the performance norms of the computerized DEM test for school-aged children in Latvia. As part of the research, the performance of the computerized DEM test for school-age children from the 1st to 6th grade was analyzed. Based on the literature sources and obtained results, selection criteria were defined, and a method was chosen for determining the norms of the computerized DEM test for school-age children in Latvia.

## 2. Materials and Methods

### 2.1. Participants

Within the framework of Latvian Council of Science project No. lzp-2021/1-0219, an extensive evaluation of visual functions and eye movements of children was carried out, including data on eye movements of children during the computerized DEM test. The study was approved by the Life and Medical Sciences Research Ethics Committee of the University of Latvia (13/06/2022). Written informed consent of the parents or legal guardians of each child was obtained prior to the enrolment in the study. A total of 378 children from 1st to 6th grade participated in the project from four different schools. The following inclusion criteria were defined for participants to perform the computerized version of the DEM test:

- Binocular single vision (assessed with the TNO test);
- No eye pathologies or general systemic diseases;
- Near visual acuity monocularly without correction at least 0.4 in decimal units (at a distance of 65 cm).

For the subsequent processing and analysis of data obtained within the study, to ensure that the results were not affected by technical factors or significant errors during the test execution, the following exclusion criteria were defined:

- Errors in eye movement recording;
- Incorrect number of rows read (rows were skipped or re-read).

In total, data from the computerized DEM test were obtained for 351 out of 378 children in all class groups—from 1st to 6th grade (see Table 1). Based on the study’s exclusion criteria, the data for 291 children were included in the further data analysis (see Table 2).

### 2.2. Equipment

The equipment used for recording eye movements during execution of the computerized DEM test was the Tobii Pro Fusion video-oculograph. Tobii Pro Fusion video-oculograph uses a stereoscopic system with two eye-tracking cameras, eye movements are recorded binocularly. The recording of the viewing position is based on the reflection of the pupil and cornea. The recording accuracy is approximately 0.3° under optimal conditions [9]. In the study, eye movement recording was conducted using the Tobii Pro Fusion eye tracker at a frequency of 250 Hz and a working distance of 65 cm.

### 2.3. Research Design

Data were collected on children’s performance on computerized and paper versions of the DEM test, initially starting with printed version and after around 10–20 min—the digital version. A standard paper version of the DEM test was applied, which consisted of two parts, a vertical part (A and B test cards) and a horizontal part (C test card). The test cards were placed at a distance of 40 cm from the subject. Font used in the test: Calibri 11 pt. Test angular size: row spacing 0.46°, digit height 0.37°, digit width 0.27°, column spacing (part A and B) 8°, the distance between digits in a row in the C card was from 1.4° to 6.7°. The time of naming the numbers of each card and the number of the errors were recorded. The DEM test performance time was adjusted for omissions and additions:

Adjusted T = T × (n/(n − o + a)),(1)

where T is the execution time (s) of the specified part of the DEM test, a—added digits, o—omitted digits, and n—the number of digits of the DEM test card.

Four parameters were defined for evaluating the performance of the paper version of the DEM test:

- Adjusted execution time of the vertical part;
- Adjusted execution time of the horizontal part;
- Ratio;
- Errors.

In the computerized version of the DEM test, a shortened C card of the DEM test was used, consisting of 40 digits (8 rows of 5 digits each). The test was displayed on a computer screen at a distance of 65 cm. The participant’s head was stabilized with a forehead and chin rest. The angular size of the computerized DEM test card C was identical to the paper version.

The study consisted of three parts. In the first part of the study, the collected data were manually processed using MathWorks MATLAB (R2020a) and MS Excel 2016 programs. The I2MC (identification by two-means clustering) algorithm was used for fixation analysis [10]. Data about X and Y coordinates (in pixels) during viewing, fixation durations (in milliseconds), fixation start and end times, and the number of fixations were obtained. The start and end positions of the test were refined and manually corrected. Additionally, the number of lines read and errors in eye movement recording were assessed from the graphical representation to verify data compliance with the established criteria.

From the data analysis of the first part of the study, data were obtained for five parameters of the computerized DEM test:

- Average duration of fixation;
- Adjusted total test (Part C) execution time (determined by subtracting the start time of the first fixation from the end time of the last fixation, and it was corrected according to Equation (1);
- Total average time spent per digit (dividing the adjusted total test (Part C) execution time by the number of digits in the computerized version of the DEM test Part C (40 digits);
- Number of fixations;
- Total number of errors (fixated manually).

In several studies on the norms of the manual DEM test 1 SD (one standard deviation) was used as the critical threshold value for the DEM test performance [11,12,13,14,15]. This means that test results falling below 1 SD from the mean can be considered age inappropriate. In the second part of the study, based on the literature sources, a criterion of 1 SD (one standard deviation) was chosen as the criterion for the computerized DEM test. Similarly, for the other four obtained parameters (average duration of fixation; adjusted total test (Part C) execution time; total average time spent per digit; number of fixations) a parameter was defined for further determination of computerized DEM test norms.

In the third part of the study, the norms for the computerized DEM test were determined for school-aged children from grades 1 to 6 in the Latvian language.

## 3. Results

From the 378 participants of the study, computerized DEM test performance data were obtained for 351 children, according to the study selection criteria (errors in eye movement recording; incorrect number of rows read), the analyzed data were for 291 children. So, the performance of the computerized DEM test obtained in the study was 77%.

The computerized DEM test version (Part C) used in the study was shorter than the manual DEM test version (Part C) consisting of 40 digits (8 rows), while the manual DEM test (Part C) consisted of 80 digits (16 rows). Therefore, the number of errors (added/omitted digits) made during the execution of both test versions was examined. The results show that 41.29% of participants did not make any errors of “added digits” in both the manual and computerized versions of the DEM test and 31.18% of participants made errors of “added digits” only in the manual version of the DEM test, 8.99% of participants made errors of “added digits” only in the computerized version of the DEM test and 18.54% of participants made errors of “added digits” in both the manual and computerized versions of the DEM test. A total of 35.11% of the participants did not make errors of “omitted digits” in both versions of the DEM test, 39.89% of the participants made errors of “omitted digits” only in the manual version of the DEM test, 3.65% of the participants made errors of “omitted digits” only in the computerized version of the DEM test, and 21.35% of the participants madeerrors of “omitted digits” in both the manual and computerized versions of the DEM test.

### 3.1. Estimation of the Parameters

**Figure 2:**
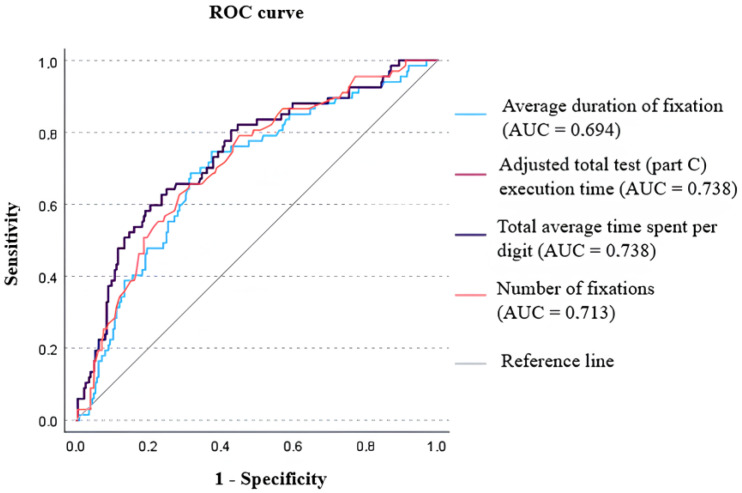
ROC curves for four parameters: average duration of fixation (ms); adjusted total test (Part C) execution time (s); total average time spent per digit (s); number of fixations. The gray line is the reference line. AUC—area under the curve.

Four parameters were examined: average duration of fixation (ms); adjusted total test (Part C) execution time (s); total average time spent per digit (s); and number of fixations. ROC analysis was used to evaluate the parameters. This method allows for estimating the accuracy of the test, regardless of the chosen threshold. Figure 2 represents the ROC curves for the four examined parameters and the area under the ROC curves (AUC). As can be seen, the ROC curves for the parameter “adjusted total test (Part C) execution time” and “total average time spent per digit” are identical (*p* = 1) and have a higher AUC value compared to the other parameters, which is 0.738. The parameters “number of fixations” and “average duration of fixation” have lower AUC values, 0.713 and 0.694, respectively. Comparing the AUC values, it was found that there was no statistically significant difference between the parameters “adjusted total test (Part C) execution time” and “number of fixations” (*p* = 0.125); “adjusted total test (Part C) execution time” and “average duration of fixation” (*p* = 0.156); and “number of fixations” and “average duration of fixation” (*p* = 0.661).

**Figure 3:**
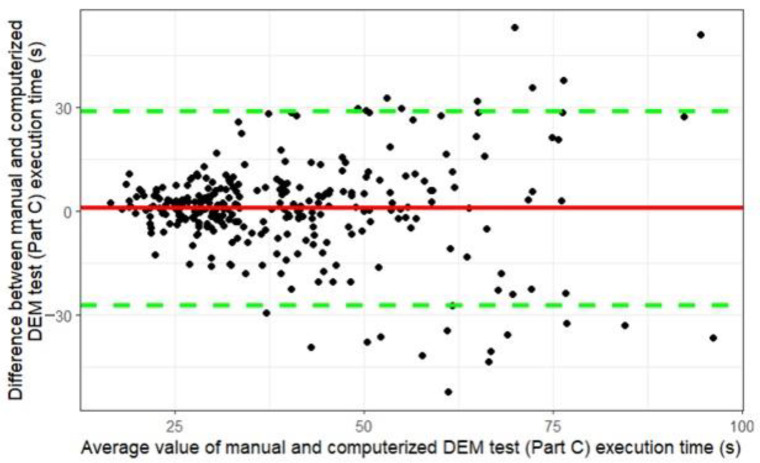
Comparison of the adjusted total DEM test (Part C) execution time between the manual and computerized DEM test versions. The black dots represent the individual results of all participants. The red line indicates the mean difference between the execution times of the manual and computerized DEM test versions; the green lines represent the upper and lower 95% confidence interval bounds for the mean difference.

A Bland–Altman analysis was used to compare manual and computerized DEM test adjusted total execution times for Part C. Since Part C of the manual version of the DEM test consisted of 80 digits (16 rows of 5 digits each), and Part C of the computerized version consisted of 40 digits (8 rows of 5 digits each), to compare the parameters, the execution time for Part C of the manual DEM test was divided by two. Results demonstrate that 93.47% of the test results for this parameter are comparable (see Figure 3). The mean difference between test execution times for the manual and computerized DEM test is 0.68 ± 14.35 s, *p* = 0.56. Indicating that participants completed the computerized test version 0.68 s faster than the manual DEM test version; however, this difference is not statistically significant. Based on the results obtained in the study, the adjusted total test (Part C) execution time was chosen as the parameter for all class groups.

### 3.2. Development of Computerized DEM Test Norms

**Figure 4:**
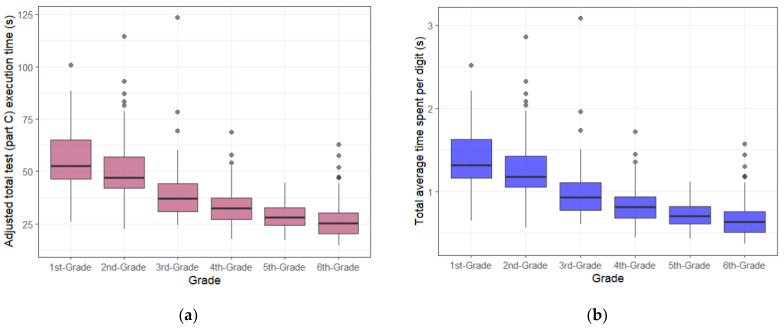

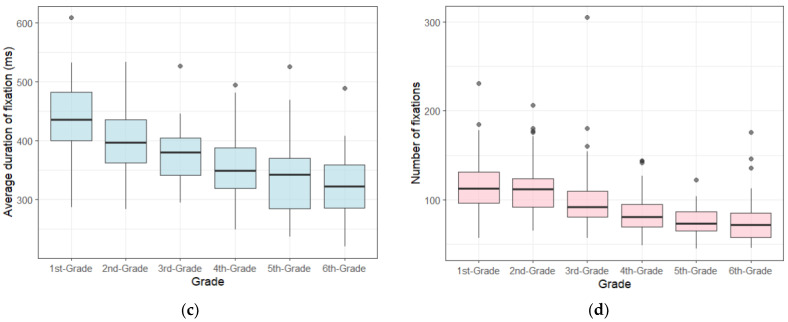
Box plot—performance results of the horizontal part (Part C) of the computerized DEM test in class groups (from 1st to 6th grade) for four parameters: (**a**) adjusted total test (Part C) execution time; (**b**) total average time spent per digit; (**c**) average duration of fixation; and (**d**) number of fixations.

Box plots were created to visualize the performance of the computerized DEM test. Figure 4 summarizes the data on the performance of all 291 children taking the computerized DEM test in each class group for the four parameters.

The norms of the computerized DEM test were calculated according to a selected criterion—one standard deviation (1 SD) and are summarized in Table 3 as minimum test performance values for four parameters (adjusted for total test (Part C) execution time; total average time spent per digit; average duration of fixation; number of fixations) in class groups from 1st to 6th grade.

## 4. Discussion

In the current study, data on children’s performance in the computerized DEM test were processed and analyzed in class groups—from 1st to 6th grade (ages 7 to 12). Four parameters obtained during the execution of the computerized DEM test were evaluated (average duration of fixation, adjusted total test (Part C) execution time, total average time spent per digit, and number of fixations). Both the manual and computerized DEM tests have a parameter called the number of errors. The calculation of the adjusted test execution time included two types of errors: added digits and omitted digits. In this study, the number of errors was taken into account in the selection criteria (high number of errors when rows were omitted or added) and was also applied as when calculating of the adjusted DEM test execution time (Equation (1)). However, the number of errors was not analyzed as a separate parameter to define the norms of the computerized DEM test. Therefore, it could be considered as another parameter to be examined more thoroughly in the future studies on both the computerized and manual DEM tests, determining the minimum acceptable number of errors for DEM test performance.

The computerized DEM test version (Part C) used in the study was shorter than the manual DEM test version (Part C) consisting of 40 digits (8 rows), while the manual DEM test (Part C) consisted of 80 digits (16 rows). A total of 31.18% of participants made the “added digits” error only in the manual DEM test version, and 39.89% of participants made the “omitted digits” error only in the manual DEM test version. The difference in the number of digits between the manual and computerized DEM test versions could be one of the factors influencing the number of errors made.

Currently, there is very little research on the norms of the computerized version of the DEM test. In several studies on the norms of the manual DEM test, 1 SD (one standard deviation) was applied as the critical value for the DEM test performance [11,12,13,14,15]. Similarly to other studies the current study also applied the 1 SD (one standard deviation) criterion to determine the norms for the computerized DEM test. The highest obtained test accuracy was 73.8%. Indicating that in future research on the computerized DEM test norms, it would be worthwhile to consider other possible critical value limits in order to find a criterion with the highest possible test accuracy.

Similar to studies on the manual version of the DEM test [12,14], this study’s results indicate that the computerized DEM test execution time of the computerized DEM test gradually decreases with age, meaning that the time required to complete the test decreases. However, the average test performance values for the 5th grade are higher than those for the 6th grade. This could be related to the presence of outliers in the 6th grade, resulting in greater data dispersion (larger standard deviation (SD)). As a result, the test parameters for the 6th grade (adjusted total test (Part C) execution time, average time spent per digit, number of fixations) have lower criteria compared to the 5th grade.

Since the current study involved eye-tracking analysis, the results of the current study provides objective data on the DEM test performance, including data on parameters that cannot be assessed with the manual version of the DEM test (i.e., the number and duration of fixations). In the study, norms of the computerized DEM test were developed in Latvian language in class groups—from 1st to 6th grade (aged 7 to 12 years), the results were summarized in a table as the minimum performance values of the computerized DEM test for four parameters (adjusted total test (Part C) execution time; total average time spent per digit; average duration of fixation; and number of fixations).

## 5. Conclusions

Evaluating the results of 1st–6th grade students in the computerized DEM test, it was concluded that the performance of the computerized DEM test is 77%.

The current study explores different parameters of the computerized DEM test and seeks to find age appropriate norms for the test performance. According to the study results, there is no statistically significant difference between the four parameters of the computerized DEM test (the test accuracy obtained in the ROC analysis (AUC value) is 73.8% for the parameter “adjusted total test (Part C) execution time”, 73.8% for the parameter “total average time spent per digit”, 69.4% for the parameter “average duration of fixation”, and 71.3% for the parameter “number of fixations”).

The parameter “adjusted total test (Part C) execution time” was demonstrated to be comparable between the manual and computerized DEM tests, and it was selected for defining the norms of the computerized DEM test.

Within the framework of the study, the norms for the computerized DEM test were determined for school-aged children in the Latvian language in class groups from 1st to 6th grade (ages 7 to 12).

The study established guidelines for developing an objective and quantitative tool for assessing children’s oculomotor and visual processing speed. The tool, designed as a screening instrument, achieved the following outcomes: (1) identifying children who perform at an age-appropriate level, (2) offering a second attempt if initial data detection fails, and (3) indicating significant difficulty if the child cannot complete the test after the second or third attempt.

## Figures and Tables

**Table 1:** Age of participants (years).

Grade	1	2	3	4	5	6
Average age ± SD	6.9 ± 0.3	7.8 ± 0.4	8.8 ± 0.4	10.0 ± 0.3	10.8 ± 0.4	12.0 ± 0.0
Age range	6–7	7–8	8–9	9–10	10–11	12

**Table 2:** Number of participants.

Grade	1	2	3	4	5	6
Number of participants	59	64	42	75	63	48
Excluded	9	16	3	12	14	6
Analyzed	50	48	39	63	49	42

**Table 3:** The mean values and standard deviations of the parameters in class groups from 1st to 6th grade, and the developed criterion (the minimum performance value) for the computerized DEM test.

Parameter		Grade	1	2	3	4	5	6
	Results							
Adjusted total test execution time (s)	Average		57	52	42	33	29	28
	SD		16	19	18	10	6	11
	**Criterion**		**73**	**71**	**60**	**43**	**35**	**39**
Total average time spent per digit (s)	Average		1.42	1.30	1.05	0.83	0.72	0.70
	SD		0.41	0.47	0.46	0.25	0.16	0.28
	**Criterion**		**1.83**	**1.77**	**1.51**	**1.08**	**0.88**	**0.98**
Average duration of fixation (ms)	Average		440	403	376	356	337	325
	SD		61	58	48	55	64	57
	**Criterion**		**501**	**462**	**424**	**412**	**401**	**382**
Number of fixations	Average		116	114	103	85	76	77
	SD		33	33	42	22	15	27
	**Criterion**		**149**	**147**	**145**	**107**	**91**	**104**

## Data Availability

The data presented in this study are available on request from the corresponding author. The data are not publicly available due to privacy reasons.
